# The role of CTGF and MFG-E8 in the prognosis assessment of SCAP: a study combining machine learning and nomogram analysis

**DOI:** 10.3389/fimmu.2025.1446415

**Published:** 2025-01-23

**Authors:** Tingting Lin, Huimin Wan, Jie Ming, Yifei Liang, Linxin Ran, Jingjing Lu

**Affiliations:** ^1^ Department of Respiratory Medicine, Xiamen Humanity Hospital, Fujian Medical University, Xiamen, China; ^2^ Shanghai East Hospital, School of Medicine, Tongji University, Shanghai, China

**Keywords:** SCAP, CTGF, MFG-E8, machine learning, nomogram

## Abstract

**Background:**

Severe Community-Acquired Pneumonia (SCAP) is a serious global health issue with high incidence and mortality rates. In recent years, the role of biomarkers such as Connective Tissue Growth Factor (CTGF) and Milk Fat Globule-Epidermal Growth Factor 8 (MFG-E8) in disease diagnosis and prognosis has increasingly gained attention. However, their specific functions in SCAP have still remained unclear. By conducting a prospective analysis, this study has explored the relationship between these two proteins and the diagnosis and mortality of SCAP patients. Additionally, founded on comparing the applications of machine learning and nomograms as predictive models in forecasting the 28-day mortality risk of SCAP patients, this paper has discussed their performance in different medical scenarios to provide more accurate treatment options and improve prognosis.

**Methods:**

198 patients diagnosed with SCAP, 80 patients with CAP and 80 healthy individuals were encompassed in the study. Demographic characteristics, clinical features and biomarkers were extracted. The ELISA method was employed to measure the levels of MFG-E8 and CTGF in the three groups. The 28-day mortality of SCAP patients was tracked. Eleven models, including XGBoost and CatBoost, were used as prediction models and compared with a nomogram. And 14 scoring methods, like F1 Score and AUC Score, were used to evaluate the prediction models.

**Results:**

Compared to healthy controls, SCAP patients had higher serum levels of CTGF and MFG-E8, suggesting that these biomarkers are associated with poor prognosis. Compared to CAP patients, SCAP patients had lower levels of MFG-E8 and higher levels of CTGF. In the deceased group of SCAP patients, their CTGF levels were higher and MFG-E8 levels were lower. Using the CatBoost model for prediction, it performed the best, with key predictive features including Oxygenation Index, cTnT, MFG-E8, Dyspnea, CTGF and PaCO2.

**Conclusion:**

This study has highlighted the critical role of clinical and biochemical markers such as CTGF and MFG-E8 in assessing the severity and prognosis of SCAP. The CatBoost model has shown the significant potential in predicting mortality risk by virtue of its unique algorithmic advantages and efficiency.

## Introduction

Community-acquired pneumonia (CAP) is a serious health concern that can lead to acute respiratory distress syndrome (ARDS) if improperly treated (1), with a mortality rate that cannot be ignored (2–6). Each year, a substantial number of adults are hospitalized due to CAP, with 10% to 20% needing ICU admission (7, 8).Despite a decline in the 30-day mortality rate for hospitalized CAP patients over the past decade (9), large-scale studies have indicated that the mortality by SCAP remains unacceptably high (10–12). Assessing the severity of the disease is a critical step in treating SCAP (13–15), because it serves as the early identification of high-risk patients aids in deciding the location and intensity of treatment and in the rational allocation of medical resources (16). Developing rapid diagnostic methods or identifying new biomarkers is crucial for mitigating the severity and mortality of SCAP.

Biomarkers, as precise and reliable biological indicators in patient samples, are essential for diagnosing pneumonia, determining its etiology, assessing risks, making triage decisions, measuring severity and guiding antibiotic treatment strategies (17, 18). For instance, Ebrahimi et al. (19) suggested that FGF21 levels reflect better pneumonia severity and 30-day mortality than traditional markers, while Liu et al. (20) have shown that elevated levels of PDGF-BB, IP-10, and RANTES can effectively distinguish various types of acute pneumonia infections.

Connective Tissue Growth Factor (CTGF), a key cytokine in the CCN family, plays a crucial role in tissue repair, fibrosis, angiogenesis, tumor progression and cell proliferation. Its interaction with cell surface receptors significantly affects cellular behaviors, including proliferation, migration, and differentiation (21–23). Abnormal expression of CTGF is associated with various diseases, such as organ fibrosis, cancer progression, and specific genetic disorders. Recent studies have stressed that miR-26a-5p can inhibit TLR signaling by suppressing CTGF expression and reducing pro-inflammatory factor expression in alveolar macrophages in mice, which has meant CTGF can serve as a potential target for predicting and treating SCAP (24). MFG-E8, a key protein found in mammalian milk fat globule membranes, is known for its diverse biological functions, such as immune modulation, apoptotic cell clearance and tissue repair. Its structure, featuring two EGF-like repeats and two Factor V/VIII C-terminal domains, is crucial for its function. The association of MFG-E8 with various diseases, such as autoimmune diseases, cancer and cardiovascular diseases, underscores its significance (25–27). Aziz et al. (28) have emphasized MFG-E8 can alleviate the severity of acute lung injury (ALI) in regulating neutrophil migration. Mice lacking MFG-E8 exhibited worsened lung injury post-LPS injection, attributed to increased neutrophil infiltration and inflammation marker production. However, administering recombinant MFG-E8 significantly mitigated this migration. This insight suggests that levels and functionality of MFG-E8 might matter in predicting the SCAP and offering new perspectives on its prognostic potential.

The pathological features of SCAP are composed of severe inflammatory responses and lung tissue damage, with changes in CTGF and MFG-E8 potentially closely related to these characteristics. However, there are no studies to prove the relationship between SCAP and CTGF or MFG-E8. The study has measured the levels of these biomarkers in the serum of SCAP patients to explore their prognostic value and understand their potential impact on patient outcomes. Establishing a clinical prediction model for the mortality of SCAP patients is essential for enhancing the accuracy of clinical decision-making, formulating personalized treatment strategies, improving patient outcomes and promoting research along with the rational allocation of resources (5).

Recently, the technological advances and rapid development of data science have made machine learning a core tool in medical research and clinical practice, especially in predicting diseases and managing patients. Machine learning models, by processing and analyzing vast datasets—including clinical indicators, medical imaging, and genetic information—can predict the onset, progression, and prognosis of diseases (29–35). In conclusion, the application of machine learning in medicine not only enhances the precision and efficiency of treatment plans but also improves patient outcomes and quality of life.

The application of nomograms in medicine has been increasingly essential, especially in today’s digital age. Although advanced technologies allow for faster, more precise calculations with more complex methodologies and larger datasets to generate more accurate and generalizable prediction models, it’s difficult to employ these models in interpretation due to their complexity, thus hindering their use by clinicians in practice. In this context, nomograms graphically represent the impact of each predictor on the outcome, providing a more specific explanation of each predictor’s effect on the outcome. Clinicians can intuitively estimate the total effect of all predictors on a given patient and then predict survival probabilities (36, 37).

Therefore, by comprehensively comparing the capabilities of traditional nomograms and machine learning technologies, this study has predicted the 28-day mortality risk of patients with SCAP. Nomograms rely on predefined clinical parameters and statistical correlations for risk assessment, typically based on datasets that analyze the correlation between specific clinical indicators and patient outcomes to predict future health outcomes (37–39). In contrast, machine learning models use algorithm-driven methods to identify complex patterns within datasets. Furthermore, with continuous improvements in algorithms and computational capabilities, machine learning techniques demonstrate tremendous potential in handling datasets, real-time data processing, and providing personalized medical recommendations (40–43). With the aim of evaluating the effectiveness of machine learning and nomogram models in practical clinical applications, we hope to enhance the accuracy of treatment strategies and ultimately clinical outcomes for patients.

## Materials and methods

This experiment utilized 6- to 8-week-old male C57BL/6 mice (20–25 g). The mice were housed under SPF conditions at the Laboratory Animal Center of Tongji University, acclimated for 7 days, and provided with standard chow and water. The mice were randomly divided into the control group and the SCAP group, with 12 mice per group. After anesthesia with pentobarbital sodium (50 mg/kg, intraperitoneally), mice in the LPS group were administered 5 mg/kg of LPS solution via intranasal instillation. LPS (from Escherichia coli O111:B4, Cat# L4391) was purchased from Sigma-Aldrich (St. Louis, MO, USA). The control group received an equal volume of sterile PBS solution. Observation time points were set at 12 hours, 24 hours, and 48 hours post-LPS administration. At each time point, mice were euthanized via excessive carbon dioxide inhalation, and peripheral blood, bronchoalveolar lavage fluid, and lung tissues were collected. Total RNA was extracted from the samples using TRIzol reagent (Invitrogen, Waltham, MA, USA), and cDNA was synthesized with a Transcriptor First Strand cDNA Synthesis Kit (Thermo Scientific, Rockford, IL, USA). Real-time quantitative polymerase chain reaction (RT-qPCR) was performed using PowerUp™ SYBR™ Green Master Mix (Thermo Scientific, Rockford, IL, USA). Gene expression levels were normalized to β-actin as the housekeeping gene and calculated using the 2^−ΔCt method. Relative gene expression was assessed using the 2^−ΔΔCt method. The experimental data were analyzed using GraphPad Prism 9 software, expressed as mean ± standard deviation. Differences between groups were assessed using one-way analysis of variance (ANOVA), with a significance level set at p < 0.05. The study passed the ethical review for animal biomedical research conducted by the Experimental Animal Center of Tongji University (TJBB03724102).

All researchers were from Shanghai East Hospital, affiliated with Tongji University. 198 patients with SCAP, 80 patients diagnosed with CAP and 80 healthy controls were encompassed in the study from July 5, 2022 to September 30, 2023. Besides, resampling techniques and cross-validation methods were employed to address the dataset imbalance. The inclusion criteria were based on the diagnosis standards for SCAP outlined in the consensus guidelines for the management of community-acquired pneumonia in adults by the Infectious Diseases Society of America/American Thoracic Society (4). The specific criteria were composed of major diagnostic criteria, such as the requirement for tracheal intubation and mechanical ventilation or septic shock requiring vasopressor therapy after adequate fluid resuscitation, and minor diagnostic criteria, including a respiratory rate > 30 breaths/min, oxygenation index (PaO2/FiO2) ≤ 250 mmHg, multilobar infiltration, altered mental status and/or disorientation, blood urea nitrogen ≥ 7.14 mmol/L, systolic blood pressure < 90 mmHg, leukopenia (WBC) < 410^9/L, thrombocytopenia (PLT) < 10010^9/L, and hypothermia < 36°C. A diagnosis of SCAP can be made if one major criterion or three or more minor criteria are met. The exclusion criteria of being under 18 years of age have received antibiotics for more than 48 hours, presence of immunodeficiency or immunosuppressive conditions such as HIV infection or post-organ transplantation, concurrent acute cardiovascular or cerebrovascular diseases and incomplete clinical data. The diagnostic criteria for CAP included community onset and pneumonia-related clinical manifestations, such as newly developed cough, sputum production, fever, signs of pulmonary consolidation and chest imaging, which can display newly emerged patchy infiltrates, lobar or segmental consolidation, ground-glass opacities, or interstitial changes, with the exclusion of other diseases like tuberculosis and lung tumors. The healthy control group was defined as individuals with no history of community-acquired pneumonia or other acute diseases, who were selected founded on their health check results to ensure they had no chronic diseases or symptoms of acute infections. Patients with SCAP and CAP in the study were primarily recruited from the hospital’s emergency department, general wards or ICU after obtaining informed consent from the patients or their families. The healthy control group, who had undergone strict screening to exclude any health issues that could affect the study results, was recruited through a health check center. To ensure comparability in baseline characteristics between the SCAP, CAP groups and healthy controls, the study employed the Propensity Score Matching (PSM) method. By calculating the propensity scores of patients at the time of inclusion, patients with similar baseline characteristics were paired with healthy individuals in terms of baseline characteristics including age, sex, medical history (such as diabetes and hypertension) and lifestyle. After obtaining written informed consent, all participants abstained from eating or drinking from midnight the day before blood collection until the blood draw. This study was approved by the Ethics Committee of Shanghai East Hospital(Research Review No. (216) of 2022).

### Enzyme-linked immunosorbent assay

Fasting blood samples were collected from participants in the morning, centrifuged at 3000 rpm, and the serum was stored in a -80°C freezer. The levels of serum CTGF and MFG-E8 cytokines were determined using the ELISA method.The ELISA kits were purchased from SHANG HAI Animalunion Biotechnology Co., Ltd (http://www.animaluni.com).

### Statistical analysis

#### Data collection and preprocessing

##### Data collection

All 198 patients diagnosed with SCAP, 80 patients with CAP and 80 healthy controls were included in the study. The levels of CTGF and MFG-E8 in 198 SCAP patients, 80 CAP patients and 80 healthy individuals were measured using enzyme-linked immunosorbent assay (ELISA), along with other clinical information (age, gender, laboratory test results, etc.). The mortality within 28 days of admission were tracked.

##### Data cleaning

Missing values were addressed, outliers were excluded and data standardization was carried out. The Z-score method was utilized to identify outliers by calculating the deviation of data points from the mean, defining those with deviations and exceeding certain threshold as outliers. Robust scaling was employed for standardization, which scaled data based on the median and interquartile range, offering enhanced robustness against outliers.

#### Feature engineering

##### Feature scaling

During the data transformation phase, numerical features were standardized to scale them to a uniform range, such as having a mean of 0 and a standard deviation of 1, with the aim of eliminating the impact of different magnitudes and promoting the efficiency and accuracy of subsequent analyses or model training.

##### Feature encoding

In this study, the dataset’s gender and baseline disease features were binarized by following these specific steps:

Gender Feature Binarization: The gender feature was converted to a binary feature, with males marked as 1, and females and unknown genders marked as 0.

Baseline Disease Feature Binarization: Baseline disease data were converted into a series of binary variables, with each disease corresponding to one variable. Presence of the baseline disease was marked as 1, and absence was marked as 0.

Radiographic Feature Binarization: Presence of features such as multilobar infiltration, atelectasis, and pleural effusion were marked as 1, and absence was marked as 0.

Clinical Symptom Feature Binarization: Presence of symptoms such as fever, cough, sputum production, dyspnea, and altered consciousness were marked as 1, and absence was marked as 0.

##### Feature selection

To avoid overfitting the training data, we employed the LASSO method for variable selection, an effective regularization technique. In LASSO regression analysis, as the penalty parameter λ increases, the coefficients of various variables decrease accordingly until some coefficients shrink to zero entirely. This process helps to simplify its structure by eliminating unnecessary covariates from the model. In practice, 3-fold cross-validation were employed to optimize the model, selecting those clinical features retained when the binomial deviance was minimized, to ensure the model’s validity and generalization ability.

#### Nomograms model construction

A clinical prediction model was constructed based on the selected features and graphically represented using a nomogram (Alignment Diagram). The nomogram is an intuitive visualization tool that uses scale lines to show the relative impact of each feature on the prediction outcome, which can be conducive to the understanding of the model’s predictions for clinicians or patients. Each feature in the model corresponds to a scale line on the nomogram, with the length and position of these lines adjusted according to the feature’s weight. By marking specific values on each scale line, users can quickly estimate an overall prediction score by connecting these points. This score can then be converted into the probability of the corresponding event occurrence. The intuitiveness of the nomogram can allow users to interpret complex statistical models and their predictions using a simple chart, without computational devices (44, 45).

### Building a machine learning model

In the current research, fifteen machine learning classification algorithms, including Naive Bayes, Logistic Regression, Decision Tree, Random Forest, Extra Trees, Bagging, Gradient Boosting, XGBoost (XGB), XGB combined with Logistic Regression (XGB+LR), CatBoost, CatBoost combined with Logistic Regression (CatBoost+LR), have been utilized to construct models from the given data. Each model underwent a systematic process of parameter optimization and evaluation to assess its fitting performance with the goal to select the most effective model for risk prediction. This optimization process incorporated a technique combining grid search with 3-fold cross-validation. This approach was used to graphically represent the Area Under the Curve (AUC) values and to observe changes in the model parameters. Subsequently, the model parameters were selected based on identifying those that align with the highest AUC score (46). Notably, the XGB+LR and CatBoost+LR models functioned by extracting leaf node indices as features. These indices were then input into the LR model for further training and prediction, following the training of the XGB and CatBoost models.

### Model training and validation

In this study, a rigorous model training and validation process were implemented to ensure the robustness and reliability of the classification models. The dataset was divided into a training set (80%) and a test set (20%). The training set was used to fit various models, including NaiveBayes, Logistic Regression, Decision Tree, Random Forest, Extra Trees, Bagging, GBDT, XGBoost, XGBoost+LR, CatBoost and CatBoost+LR. To optimize model parameters and mitigate overfitting, 4-fold cross-validation within the training subset were employed. This technique involved splitting the training set into four smaller subsets, training the model on three of these subsets, validating it on the remaining subset and iterating through all four subsets. The performance metric AUC was calculated and averaged over the four iterations to provide a robust estimate of model performance. To address potential class imbalance, the SMOTE (Synthetic Minority Over-sampling Technique) was applied. The validation set, independent of the training data, was used for fine-tuning model hyperparameters. The final model was selected based on its performance during cross-validation and validation set optimization, and then evaluated on the test set (20%) to assess its generalization ability and readiness for practical application. This systematic approach ensured the selection of effective classifiers for our predictive task, thus highlighting the practical utility of the models.

### Assessment of models

This paper has used common evaluation metrics in statistics and machine learning to provide different insights into the performance of the model, namely Precision, Recall, Accuracy, F1 Score, AUC Score, Youden Index, AUC 95% Confidence Interval, AUC Standard Error, Sensitivity, Specificity, Positive Predictive Value, Negative Predictive Value, Positive Likelihood Ratio, and Negative Likelihood Ratio.

### Model explainability

SHAP (Shapley Additive exPlanations) values are a method based on the Shapley values from game theory, which is used to explain the predictions of machine learning models. They provide an intuitive explanation of the model’s decisions by quantifying the contribution of each feature to the model’s prediction. Each SHAP value represents the contribution of a specific feature value relative to the baseline or average prediction. This method is applicable to various types of machine learning models to offer consistent interpretation, allowing for clear understanding of each feature’s contribution to the model’s prediction. In the context of determining survival or mortality, SHAP values can help explain how each feature influences the model’s prediction of an individual’s survival probability (47).

### Statistics and software

All statistical analyses and model training processes in this study were conducted in Python (version 3.9) and R (version 3.6.1). The ‘pandas’ and ‘numpy’ packages were utilized for data preprocessing, with the ‘pingouin’ package for t-tests and correlation analyses, the ‘imbalanced-learn’ package to implement the SMOTE algorithm and packages such as ‘sklearn’ and ‘xgboost’ for model implementation. The ‘shap’ package was used for model interpretation, while ‘matplotlib’ and ‘seaborn’ were employed for data visualization processing.

## Results

### Increased mRNA expression of CTGF and MFG-E8 correlates with pathological changes in SCAP

**Figure 1:**
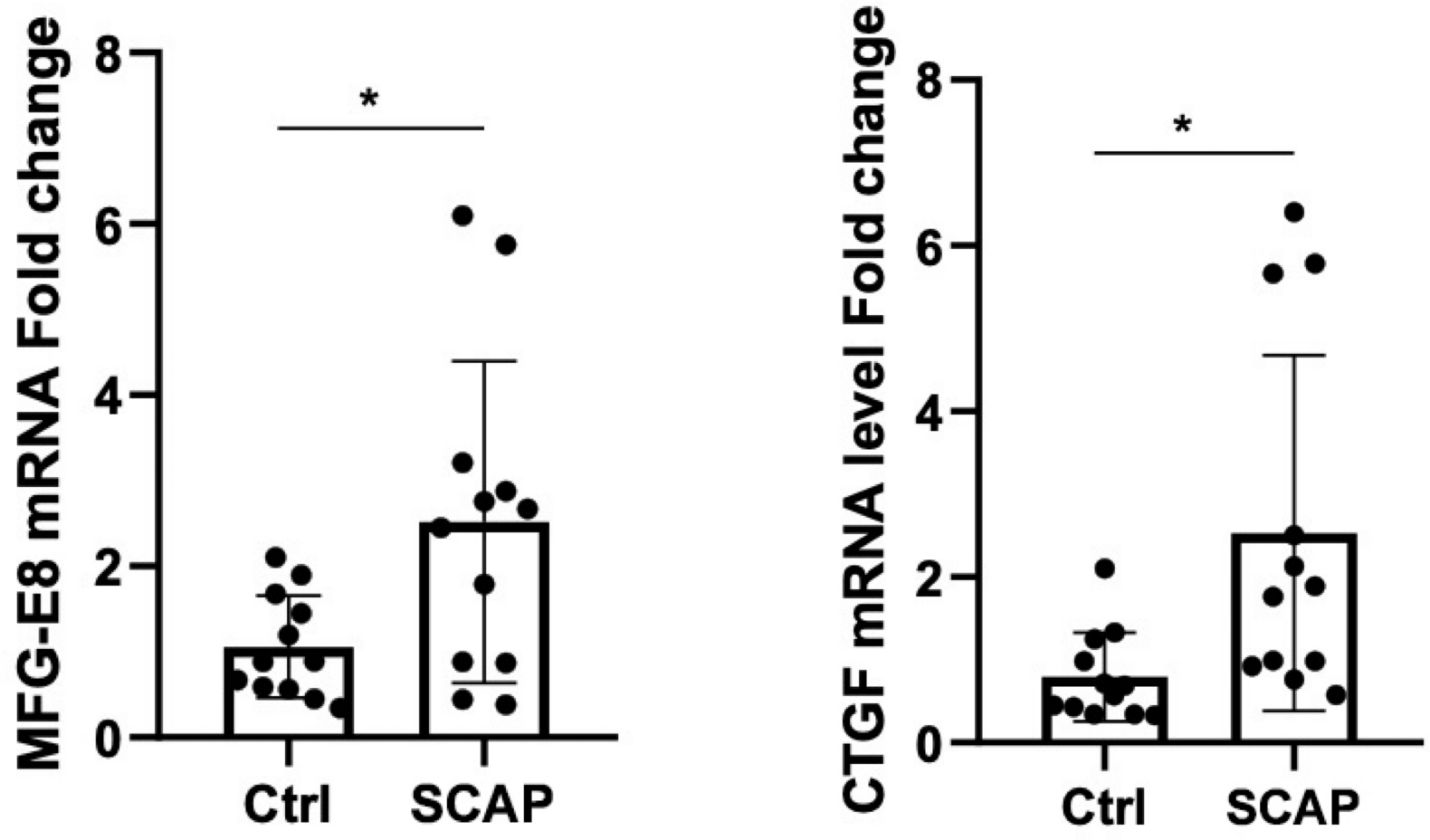
The effect of SCAP on MFG-E8 and CTGF gene expression levels. * : P<0.05.

**Figure 2:**
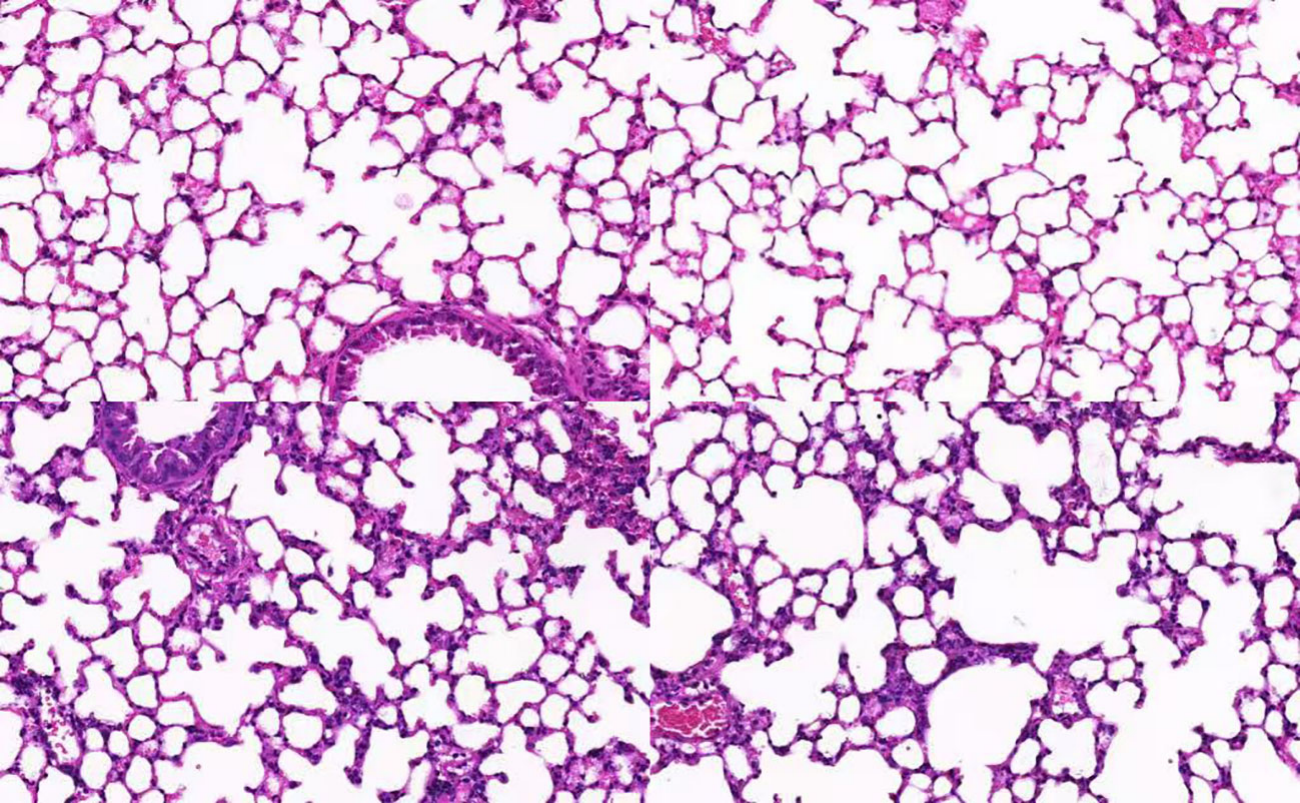
HE-Stained image of lung tissue section.

The mRNA expression levels of CTGF and MFG-E8 in peripheral blood were significantly upregulated in the SCAP group compared to the control group (p < 0.05). (**Figure 1**) Histological analysis of lung tissue through hematoxylin and eosin (H&E) staining revealed marked pathological changes in the pneumonia group, including thickened alveolar walls, inflammatory cell infiltration, edema, and partial structural destruction, whereas the control group maintained normal alveolar architecture with minimal inflammatory changes (**Figure 2**).

### Demographic characteristics and clinical information

All 198 SCAP patients, 80 CAP patients and 80 control cases were included in this study for analysis. (**Table 1**) Among them, there were 122 male SCAP patients and 76 female SCAP patients, with 157 survivors and 41 fatalities. The average age of SCAP patients was 76.13 years. Among the CAP patients, there were 42 males and 38 females, with an average age of 78.02 years, while the control group had an average age of 73.61 years, comprising 46 males and 34 females. In the detailed comparison between the SCAP, CAP and control groups, the SCAP group exhibited elevated WBC counts with an average value of 8.06 ± 3.8910^9, whereas the control group had a lower average value of 6.25 ± 2.1810^9. In addition, the SCAP group with significantly lower PaO2 levels indicated markedly reduced oxygenation efficiency. Elevated levels of various interleukins (IL-1β, IL-2, IL-4, etc.) in the SCAP group further emphasized a more intense inflammatory response, potentially reflecting deeper or more extensive inflammation. Furthermore, increased AST, ALT and creatinine levels in the SCAP group suggested more pronounced tissue damage and potential renal dysfunction, meaning a higher degree of organ distress or injury in this group.

**Table 1:** Demographic and clinical indicators of SCAP patients, CAP patients, and healthy controls.

Variables	SCAP(n=198)	CAP (n=80)	Control(n=80)
Male, n(%)	122(61.62%)	42(52.5%)	46 (56.50%)
Death, n(%)	31(20.71%)	0	0
Age(years)	76.13 ± 11.30	78.02 ± 9.73	73.16 ± 6.78
WBC(*10^9)	8.06 ± 3.89	9.38 ± 4.08	6.25 ± 2.18
Percentage_Neutrophils(%)	76.46 ± 12.77	77.9 ± 9.7	62.71 ± 12.31
Neutrophil(*10^9/L)	6.46 ± 3.65	7.58 ± 3.98	4.32 ± 3.10
Percentage_Lymphocytes(%)	15.22 ± 10.44	13.97 ± 7.02	26.79 ± 11.29
Lymphocytes(*10^9/L)	1.02 ± 0.72	1.15 ± 0.5	1.59 ± 0.69
SAA(mg/L)	169.8 ± 134.83	172.2 ± 125.7	37.20 ± 14.88
CRP(mg/L)	65.98 ± 70.89	72.51 ± 77	12.38 ± 20.71
PCT(ng/L)	0.71 ± 3.01	0.3 ± 0.56	0.15 ± 0.59
IL-1β(pg/ml)	5.34 ± 13.56	<2.5	<2.5
IL-2(pg/ml)	5.09 ± 13.22	<2.5	<2.5
IL-4(pg/ml)	4.75 ± 10.12	<2.5	<2.5
IL-5(pg/ml)	<2.5	<2.5	<2.5
IL-6(pg/ml)	10.44 ± 304.74	27.71 ± 55.31	26.83 ± 12.68
IL-8(pg/ml)	61.12 ± 105.25	24.71 ± 75.34	43.51 ± 39.41
IL-10(pg/ml)	6.2 ± 12.74	3.23 ± 2.37	4.44 ± 8.63
IL-12P70(pg/ml)	4.75 ± 19.96	<2.5	<2.5
IL-17(pg/ml)	10.85 ± 21.37	<2.5	10.17 ± 9.12
IFNα(pg/ml)	5.77 ± 10.83	<2.5	<2.5
IFNγ(pg/ml)	4.14 ± 4.65	<2.5	3.61 ± 4.84
TNGα(pg/ml)	7.11 ± 22.47	3.94 ± 3.24	<2.5
PaO2(mmHg)	87.99 ± 34.30	88.68 ± 21.56	103.72 ± 21.25
SaO2(%)	98.54 ± 61.13	97.14 ± 2.08	98.99 ± 31.36
PaCO2(mmHg)	39.15 ± 8.84	37.98 ± 6.57	40.68 ± 10.39
BE(mmol/l)	-0.14 ± 3.23	1.34 ± 16.7	-0.71 ± 1.98
cTnT(ng/ml)	0.039 ± 0.14	0.017 ± 0.017	0.01 ± 0.10
D-D(mg/L)	4.08 ± 9.94	0.92 ± 1.09	0.88 ± 1.02
CTGF(pg/ml)	322.60 ± 148.64	274.19 ± 96.37	269.16 ± 91.27
MFG-E8(pg/ml)	330.30 ± 32.58	369.20 ± 36.55	309.72 ± 16.38
AST(U/L)	45.83 ± 93.40	38.52 ± 22.44	28.05 ± 24.21
ALT(U/L)	36.14 ± 42.91	32.48 ± 27.90	31.95 ± 35.12
Lac(mmol/L)	2.52 ± 1.25	1.29 ± 0.54	1.57 ± 0.89
Cr(umol/L)	94.57 ± 76.30	81.35 ± 72.35	74.61 ± 20.80

### Comparison of serum CTGF and MFG-E8 Levels among healthy controls, SCAP patients and CAP patients

**Figure 3:**
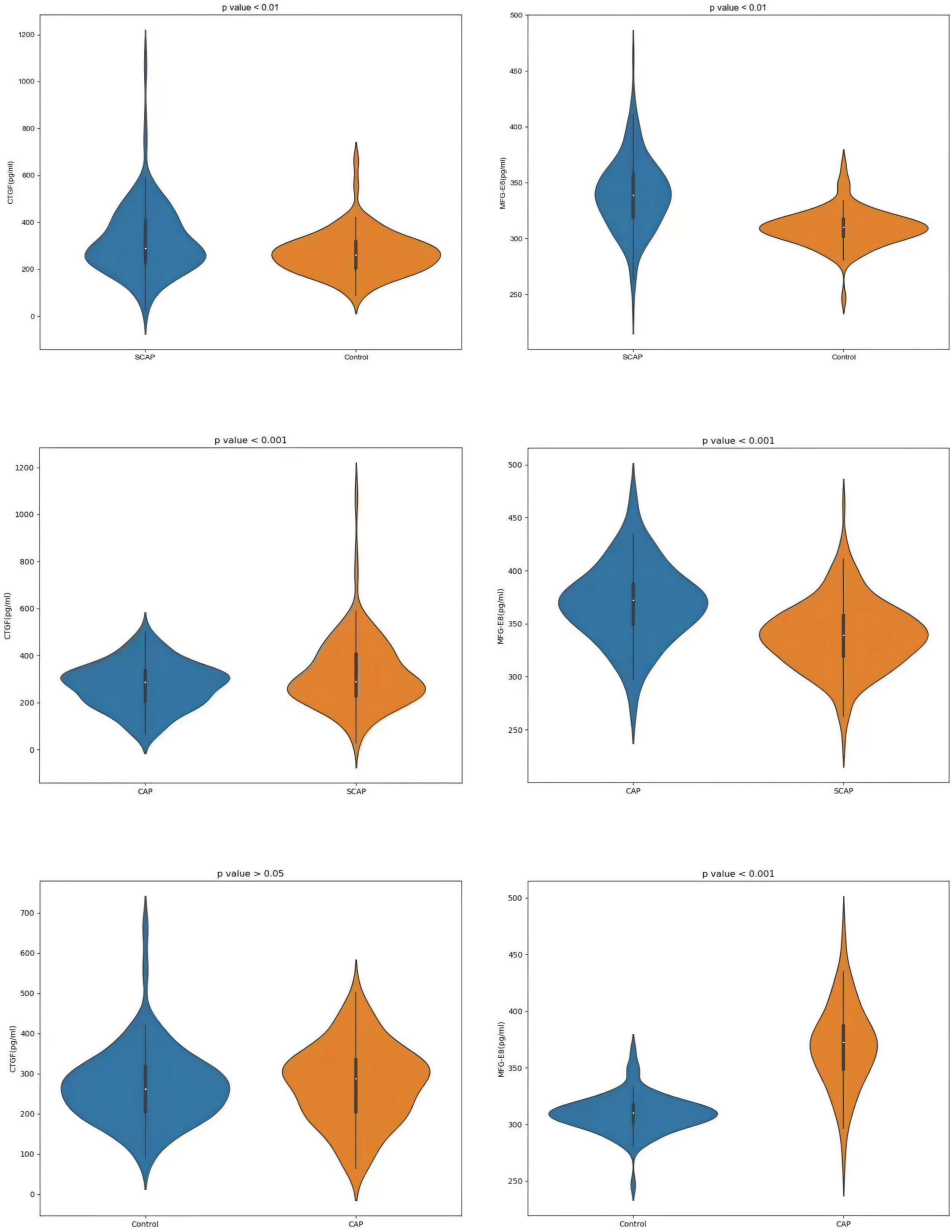
Comparison of CTGF and MFG-E8 levels among SCAP, CAP, and control groups.

In the serum of healthy controls and SCAP patients, MFG-E8 and CTGF levels were detected, both of which displayed significant differences with p-values < 0.01 (**Figures 3A, B**). The concentration of CTGF in SCAP patients was 322.60 ± 148.64 pg/ml, while the average level in healthy controls was 269.16 ± 91.27 pg/ml. Regarding MFG-E8 levels, SCAP patients had an average level of 339.30 ± 32.58 pg/ml, compared to an average of 309.72 ± 16.38 pg/ml in healthy individuals. These findings may provide new biomarkers for future diagnosis and treatment because they were significant for understanding the impact of SCAP on biomarker levels in patients.

For the CAP group, the data showed an average CTGF concentration of 274.19 ± 96.37 pg/ml and an average MFG-E8 concentration of 369.20 ± 36.55 pg/ml. On the other hand, in the SCAP group, the average concentration of CTGF was 322.60 ± 148.64 pg/ml and MFG-E8 was 330.30 ± 32.58 pg/ml. The p-values for CTGF and MFG-E8 in both the CAP and SCAP groups were less than 0.001, suggesting that these two biomarkers may be related to the severity of pneumonia (**Figures 3C, D**). In the CAP and control groups, the P value for CTGF is less than 0.05, while the P value for MFG-E8 is less than 0.001 (**Figures 3E, F**).

### Univariate analysis of case data between survival and mortality groups and logistic regression analysis of factors influencing mortality in SCAP patients

This study conducted a detailed univariate statistical assessment of 198 SCAP patients, including survivors (n=157) and non-survivors (n=41). T-tests, Mann-Whitney tests and chi-square tests were used to make statistical comparisons. Significant differences between the two groups that were found in AGE, MFG-E8, CTGF, Percentage Neutrophils, Lymphocytes, SAA, CRP, PCT, PaO2, SaO2, PaCO2, cTnT, D-D, CTGF, AST, Oxygenation Index and Lac indicated their potential relevance in predicting survival outcomes (**Table 2**).

**Table 2:** Single factor comparison table of survival status between the survival group and death group of SCAP patients.

Variables	Survival (n = 157)	Death (n = 41)	Statistic	*P*
AGE, Mean ± SD	74.89 ± 11.64	80.88 ± 8.64	t=-3.66	<.001
MFG-E8(pg/ml), Mean ± SD	343.70 ± 31.26	322.46 ± 32.83	t=3.83	<.001
Oxygenation Index, Mean ± SD	344.70 ± 31.26	327.46 ± 32.83	t=3.11	0.002
Systolic blood pressure (mmHg), Mean ± SD	134.73 ± 19.82	136.17 ± 18.94	t=-0.42	0.675
WBC(*10^9/L), M (Q_1_, Q_3_)	7.27 (4.78, 9.83)	7.59 (6.30, 10.12)	Z=-1.32	0.188
Percentage Neutrophils(%), M (Q_1_, Q_3_)	76.50 (67.20, 86.00)	83.60 (77.80, 89.80)	Z=-3.33	<.001
Neutrophil(*10^9/L), M (Q_1_, Q_3_)	5.79 (3.39, 8.13)	6.38 (5.13, 8.68)	Z=-1.94	0.052
Percentage Lymphocytes(%), M (Q_1_, Q_3_)	14.70 (8.00, 22.60)	8.70 (5.00, 12.30)	Z=-4.12	<.001
Lymphocytes(*10^9/L), M (Q_1_, Q_3_)	0.91 (0.65, 1.38)	0.62 (0.45, 0.83)	Z=-3.60	<.001
SAA(mg/L), M (Q_1_, Q_3_)	169.82 (29.75, 275.95)	236.98 (153.36, 288.00)	Z=-2.29	0.022
CRP(mg/L), M (Q_1_, Q_3_)	36.46 (12.08, 78.65)	70.17 (37.97, 116.13)	Z=-3.06	0.002
PCT(ng/L), M (Q_1_, Q_3_)	0.07 (0.04, 0.20)	0.19 (0.10, 0.56)	Z=-4.25	<.001
IL-1β(pg/ml), M (Q_1_, Q_3_)	2.50 (2.50, 5.34)	2.50 (1.25, 3.72)	Z=-0.86	0.392
IL-2(pg/ml), M (Q_1_, Q_3_)	2.50 (1.25, 5.09)	2.50 (1.25, 3.81)	Z=-0.87	0.385
IL-4(pg/ml), M (Q_1_, Q_3_)	3.05 (2.50, 4.75)	2.57 (2.50, 4.75)	Z=-0.36	0.721
IL-5(pg/ml), M (Q_1_, Q_3_)	2.35 (1.25, 2.50)	2.35 (1.25, 2.50)	Z=-0.63	0.528
IL-6(pg/ml), M (Q_1_, Q_3_)	17.40 (5.48, 100.44)	44.84 (10.88, 100.44)	Z=-1.82	0.069
IL-8(pg/ml), M (Q_1_, Q_3_)	41.61 (12.42, 61.12)	39.90 (23.10, 61.12)	Z=-0.37	0.715
IL-10(pg/ml), M (Q_1_, Q_3_)	4.05 (2.50, 6.21)	5.63 (3.34, 6.21)	Z=-1.72	0.086
IL-12P70(pg/ml), M (Q_1_, Q_3_)	2.50 (1.25, 3.08)	2.50 (1.25, 3.70)	Z=-0.02	0.983
IL-17(pg/ml), M (Q_1_, Q_3_)	8.56 (2.50, 12.79)	10.85 (5.00, 15.67)	Z=-1.22	0.222
IFNα(pg/ml), M (Q_1_, Q_3_)	2.75 (2.50, 5.77)	2.50 (2.50, 5.77)	Z=-0.98	0.329
IFNγ(pg/ml), M (Q_1_, Q_3_)	3.71 (2.50, 4.25)	4.14 (2.50, 4.22)	Z=-0.54	0.592
TNGα(pg/ml), M (Q_1_, Q_3_)	3.06 (2.50, 5.91)	3.90 (2.50, 6.53)	Z=-0.23	0.819
PaO2(mmHg), M (Q_1_, Q_3_)	84.77 (68.49, 109.80)	72.20 (59.50, 82.00)	Z=-3.69	<.001
SaO2(%), M (Q_1_, Q_3_)	97.00 (94.40, 98.60)	94.70 (91.80, 96.70)	Z=-3.34	<.001
PaCO2(mmHg), M (Q_1_, Q_3_)	38.86 (35.48, 43.40)	34.60 (31.80, 37.40)	Z=-4.53	<.001
BE, M (Q_1_, Q_3_)	-0.14 (-1.20, 2.10)	0.00 (-2.40, 1.00)	Z=-1.10	0.270
Kl-6(U/Ml), M (Q_1_, Q_3_)	1041.27 ± 753.31	1024.74 ± 175.15	Z=-1.32	0.188
cTnT(Ng/Ml), M (Q_1_, Q_3_)	0.01 (0.01, 0.02)	0.03 (0.02, 0.05)	Z=-5.20	<.001
D-D(mg/L), M (Q_1_, Q_3_)	1.00 (0.44, 1.72)	2.52 (1.14, 6.45)	Z=-4.46	<.001
CTGF(pg/ml), M (Q_1_, Q_3_)	279.57 (227.75, 388.51)	303.86 (267.78, 471.58)	Z=-2.28	0.023
AST(U/L), M (Q_1_, Q_3_)	31.00 (24.00, 43.00)	41.00 (31.10, 56.10)	Z=-2.69	0.007
ALT(U/L), M (Q_1_, Q_3_)	25.00 (19.00, 38.00)	30.00 (24.00, 46.00)	Z=-1.68	0.092
Lac(mmol/L), M (Q_1_, Q_3_)	2.30 (1.70, 2.90)	2.50 (2.10, 3.50)	Z=-2.20	0.028
Cr(umol/L), M (Q_1_, Q_3_)	75.00 (60.00, 91.00)	87.70 (69.00, 127.00)	Z=-2.67	0.008
Respiratory rate (times/minute), M (Q_1_, Q_3_)	20.00 (18.00, 21.00)	19.00 (18.00, 21.00)	Z=-1.38	0.167
Urea nitrogen (mmol/L), M (Q_1_, Q_3_)	8.25 (4.78, 12.83)	9.00 (4.76, 12.11)	Z=-0.24	0.809
Sex, n(%)			χ²=4.28	0.039
Female	66 (42.04)	10 (24.39)		
Man	91 (57.96)	31 (75.61)		
Coronary Heart Disease, n(%)			χ²=2.60	0.107
0	132 (84.08)	30 (73.17)		
1	25 (15.92)	11 (26.83)		
Chronic Bronchitis, n(%)			χ²=0.58	0.447
0	151 (96.18)	41 (100.00)		
1	6 (3.82)	0 (0.00)		
COPD, n(%)			χ²=0.56	0.456
0	149 (94.90)	37 (90.24)		
1	8 (5.10)	4 (9.76)		
Atrial Fibrillation, n(%)			χ²=0.00	1.000
0	149 (94.90)	39 (95.12)		
1	8 (5.10)	2 (4.88)		
Diabetes, n(%)			χ²=0.39	0.534
0	99 (63.06)	28 (68.29)		
1	58 (36.94)	13 (31.71)		
Hypertension, n(%)			χ²=0.19	0.662
0	63 (40.13)	18 (43.90)		
1	94 (59.87)	23 (56.10)		
Fever, n(%)			χ²=2.47	0.116
0	32 (20.38)	4 (9.76)		
1	125 (79.62)	37 (90.24)		
Cough, n(%)			χ²=0.04	0.835
0	15 (9.55)	5 (12.20)		
1	142 (90.45)	36 (87.80)		
Dyspnea, n(%)			χ²=2.89	0.089
0	84 (53.50)	28 (68.29)		
1	73 (46.50)	13 (31.71)		
Disturbance of consciousness, n(%)			χ²=5.77	0.016
0	133 (84.71)	41 (100.00)		
1	24 (15.29)	0 (0.00)		
Multiple lobar infiltration, n(%)			χ²=1.76	0.185
0	41 (26.11)	15 (36.59)		
1	116 (73.89)	26 (63.41)		
Atelectasis of the lungs, n(%)			χ²=0.01	0.931
0	141 (89.81)	36 (87.80)		
1	16 (10.19)	5 (12.20)		
Pleural effusion, n(%)			χ²=0.17	0.679
0	94 (59.87)	26 (63.41)		
1	63 (40.13)	15 (36.59)		

**Table 3** presents the results of the logistic regression analysis, which was used to assess the factors influencing the risk of mortality in patients. The analysis showed that indicators such as CTGF, MFG-E8 and Cr displayed significant potential as biomarkers for predicting the risk of mortality in SCAP patients and risk factors affecting patient mortality.

**Table 3:** Logistic regression analysis table for mortality of SCAP patients.

Variables	β	S.E	Z	*P*	OR (95%CI)
AGE	0.05	0.02	2.97	0.003	1.06 (1.02 ~ 1.09)
Percentage Neutrophils(%)	0.06	0.02	3.19	0.001	1.06 (1.02 ~ 1.10)
Percentage Lymphocytes(%)	-0.11	0.03	-3.75	<.001	0.90 (0.85 ~ 0.95)
Lymphocytes(*10^9/L)	-1.28	0.44	-2.90	0.004	0.28 (0.12 ~ 0.66)
SAA(mg/L)	0.00	0.00	1.47	0.141	1.00 (1.00 ~ 1.00)
CRP(mg/L)	0.01	0.00	2.12	0.034	1.01 (1.01 ~ 1.01)
PCT(ng/L)	0.01	0.06	0.09	0.926	1.01 (0.90 ~ 1.12)
PaO2(mmHg)	-0.03	0.01	-3.45	<.001	0.97 (0.96 ~ 0.99)
SaO2(%)	0.00	0.00	1.07	0.284	1.00 (1.00 ~ 1.01)
PaCO2(mmHg)	-0.11	0.03	-3.31	<.001	0.90 (0.84 ~ 0.96)
cTnT(Ng/Ml)	1.85	1.18	1.57	0.116	6.35 (0.63 ~ 63.59)
D-D(mg/L)	0.04	0.02	2.31	0.021	1.04 (1.01 ~ 1.08)
MFG-E8(pg/ml)	-0.04	0.01	-4.41	<.001	0.96 (0.94 ~ 0.98)
CTGF(pg/ml)	0.01	0.00	3.14	0.002	1.01 (1.01 ~ 1.01)
AST(U/L)	0.01	0.01	1.06	0.289	1.01 (0.99 ~ 1.02)
Lac(mmol/L)	0.35	0.14	2.55	0.011	1.41 (1.08 ~ 1.84)
Cr(umol/L)	0.01	0.00	2.77	0.006	1.01 (1.01 ~ 1.01)
Oxygenation Index	-0.02	0.01	-2.98	0.003	0.98 (0.97 ~ 0.99)
Sex					
Female					1.00 (Reference)
Man	0.81	0.40	2.04	0.042	2.25 (1.03 ~ 4.90)
Disturbance of consciousness					
0					1.00 (Reference)
1	-17.40	1304.53	-0.01	0.989	0.00 (0.00 ~ Inf)
Multiple lobar infiltration					
0					1.00 (Reference)
1	2.20	1.03	2.13	0.033	9.06 (1.20 ~ 68.65)

### Correlation analysis of various features with SCAP mortality

**Figure 4:**
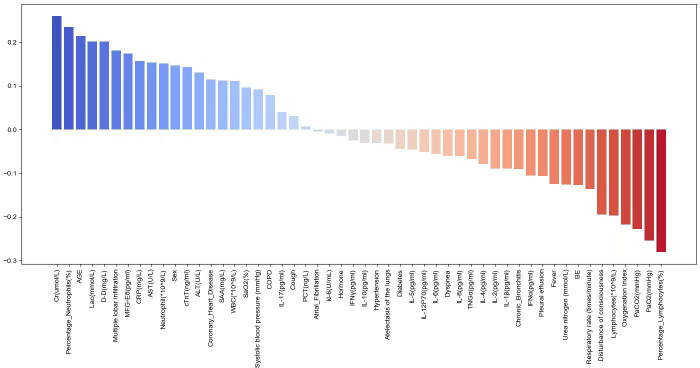
Pearson coefficient plot.

The study evaluated the correlation of various features with SCAP mortality using the Spearman correlation coefficient. **Figure 4** illustrates the Pearson correlation coefficients to show the strength of the relationship between various biomarkers and the risk of mortality in SCAP patients. The length of each bar quantitatively reflects the degree of correlation between each variable and patient outcomes. Blue bars indicating a positive correlation suggest that as the levels or presence of these factors increase, the risk of mortality also increases. These factors included Cr, percentage of Neutrophils, AGE, Lac, D-D, CTGF, and CRP. Conversely, red bars meaning an inverse relationship show an increase in these factors, which is associated with a lower risk of mortality. These factors encompass the percentage of Lymphocytes, MFG-E8, PaO2, PaCO2, Lymphocytes and Disturbance of consciousness.

### Comparison of ROC analysis for MFG-E8 and CTGF in predicting mortality risk in SCAP patients

**Figure 5:**
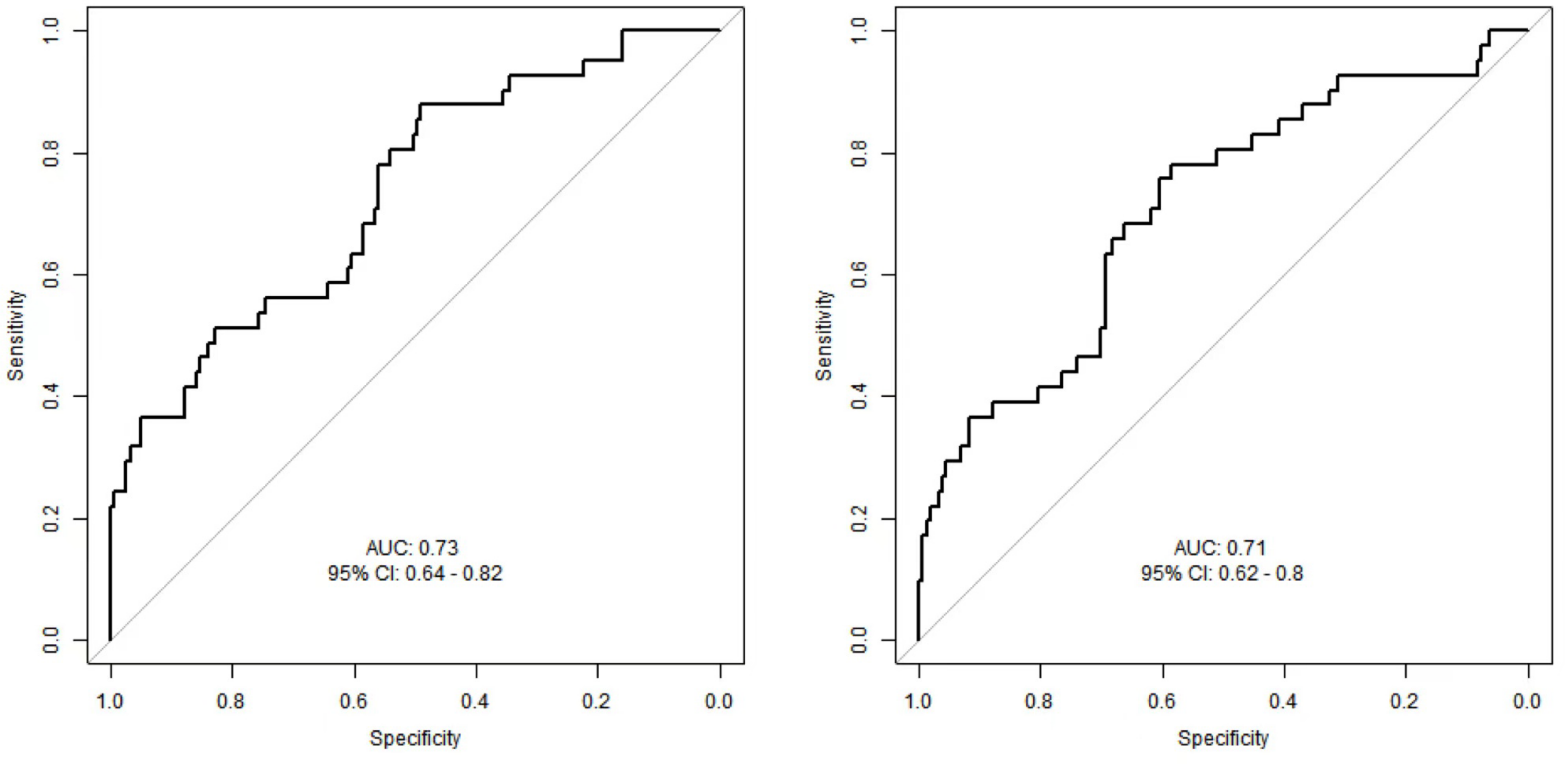
ROC curves for predicting mortality risk in SCAP patients.

The ROC curves (**Figures 5A, B**) illustrate the performance of MFG-E8 and CTGF in predicting mortality risk in SCAP patients. The AUC for MFG-E8 is 0.73, with a 95% confidence interval (CI) of 0.64 - 0.82 and the AUC for CTGF is 0.71, with a 95% CI of 0.62 - 0.8. By comparing the two ROC curves, MFG-E8 shows slightly better predictive capability than CTGF, but neither is sufficient on its own to predict the 28-day mortality outcome in SCAP patients. In practical applications, the ultimate utility of these biomarkers may require their combination with other clinical indicators and information to enhance predictive accuracy, thereby providing more information for the treatment and management of SCAP patients.

### Feature selection and nomogram model establishment

**Figure 6:**
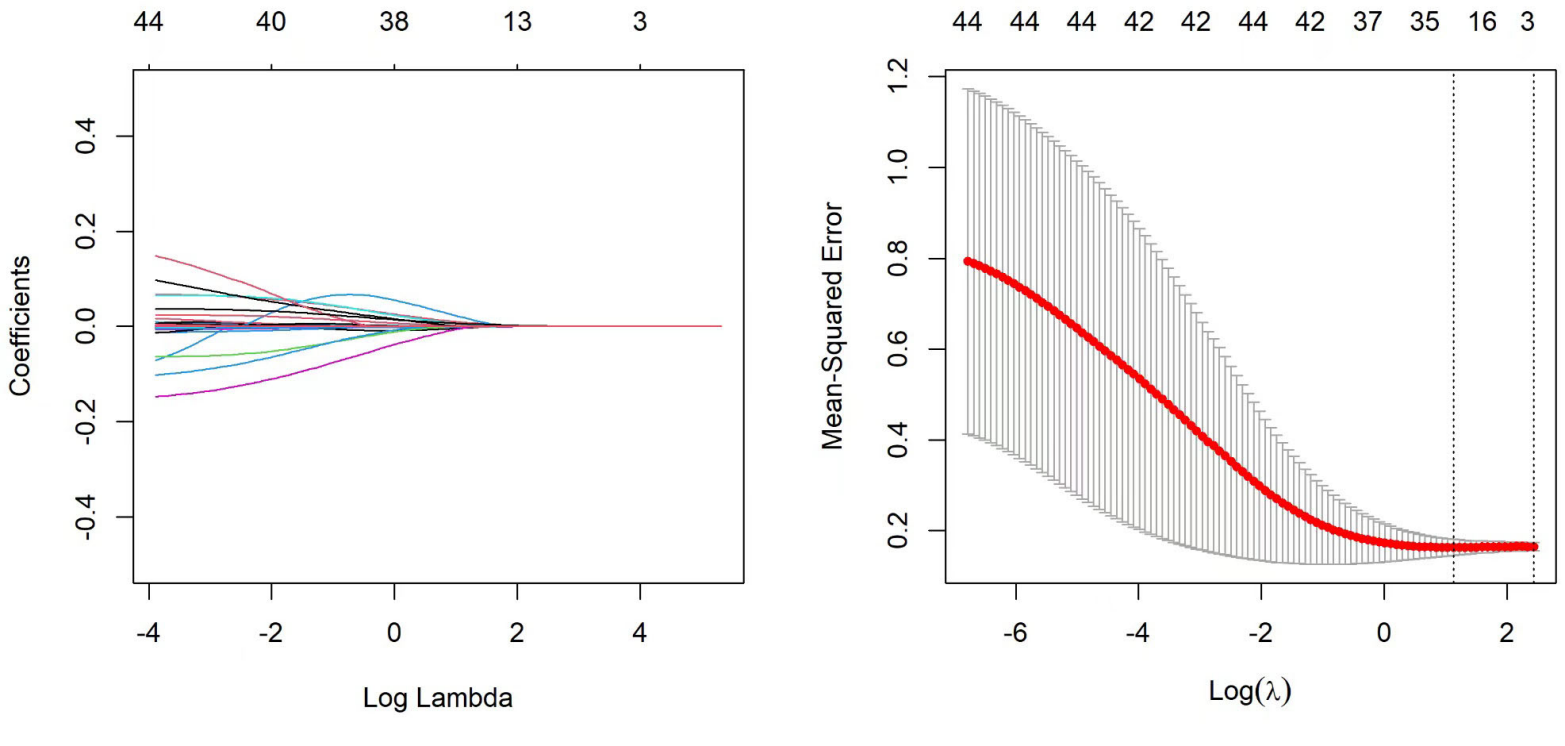
Lasso regression analysis Lasso.

**Figure 7:**
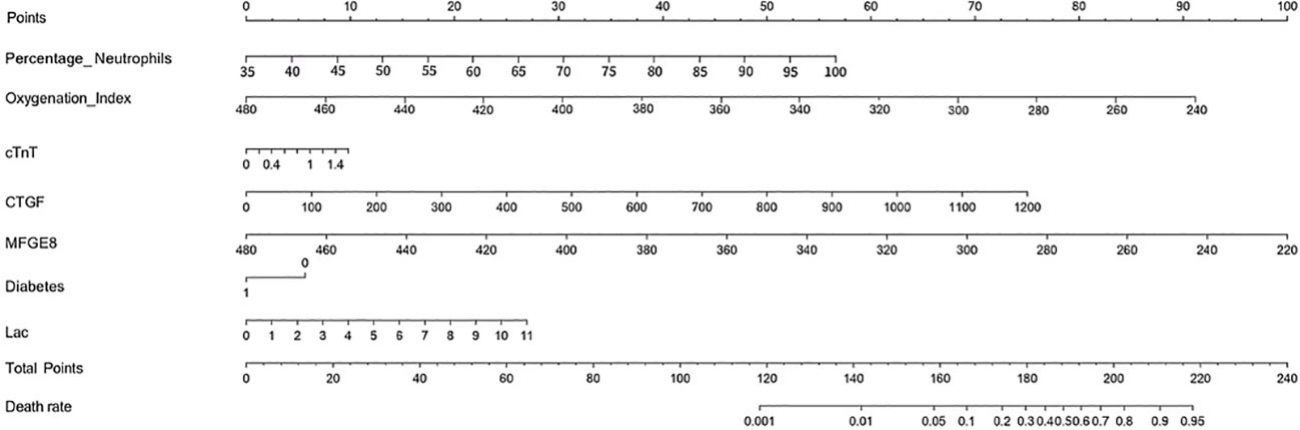
Column chart of predictive model for mortality risk in SCAP patients.

A total of 52 variables are included in our feature selection process. The results of the LASSO regression are depicted in **Figures 6A, B**. **Figure 6A** illustrates the gradual reduction of coefficients for each variable as the penalty coefficient increases until reaching zero, while **Figure 6B** demonstrates the minimum binomial deviation. Through Lasso analysis, seven statistically significant variables, CTGF, cTnT, MFGE8, Diabetes, Oxygenation Index, Percentage Neutrophils, and Lac (**Table 4**), have been selected. These seven variables were then incorporated into the construction of the predictive model. A nomogram for the predictive model was established using R to predict whether SCAP patients would survive or not, as shown in **Figure 7**. The top row of the figure represents the scale for estimating the risk score for each variable. The corresponding score for each variable value can be read on the Points scale vertically, and then the total score is calculated on the Total Points scale. Finally, the SCAP mortality risk can be determined based on the value corresponding to the total score on the bottom row.

**Table 4:** Obtaining optimal clinical features of SCAP through dimensionality reduction using LASSO algorithm.

Feature	Lasso coefficient
CTGF	1.30293911
cTnT	0.82086296
MFG-E8	-0.73542844
Diabetes	-0.12702843
Oxygenaation Index	-0.08216541
Percentage Neutrophils	0.06493113
Lac	0.02201748

### Model discrimination and evaluation using nomogram

**Figure 8:**
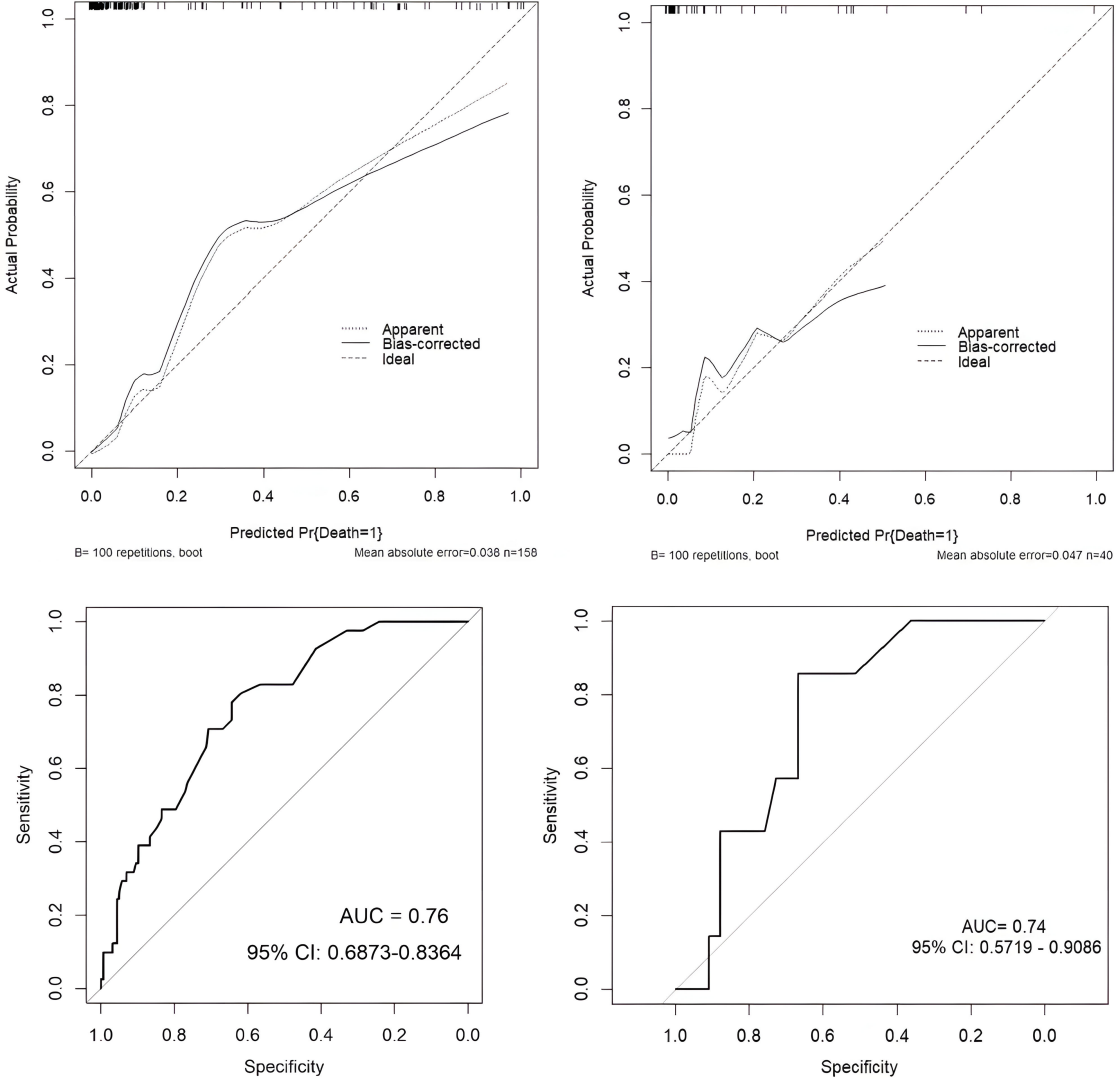
Model performance evaluation in training and test sets.

The dataset was divided into a training set of 158 individuals and a validation set of 40 individuals. Calibration curves and ROC curves were used to evaluate the accuracy and discrimination ability of the predictive model. The closer the “calibration correction” line is to the “ideal” line, the better the calibration effect of the model. **Figures 8A, B** indicate this model has good calibration. Additionally, 14 evaluation metrics—Precision, Recall, Accuracy, F1 score, AUC score, AUC 95% confidence interval, AUC standard error, Youden index, Sensitivity, Specificity, Positive predictive value, Negative predictive value, Positive likelihood ratio, and Negative likelihood ratio (**Table 5**), were employed to comprehensively evaluate the model. The results have shown that the AUC value of the training set for the predictive model is 0.76 (**Figure 8C**), indicating good predictive ability. Next, the test set was used to further examine the accuracy and discrimination ability of the model. The AUC value corresponding to the ROC curve in the test group is 0.74 (**Figure 8D**), which is similar to the results obtained in the training set. This further demonstrates the high accuracy and reliability of the predictive model we constructed for predicting the mortality rate of SCAP patients.

**Table 5:** Table of performance evaluation metrics for nomogram model.

Scoring indicators	Score
Precision	0.500
Recall	0.024
Accuracy	0.793
F1score	0.665
AUC score	0.764
AUC 95% confidence interval	0.687-0.836
AUC standard error	0.077
Youden index	0.018
Sensitivity	0.994
Specifity	0.024
Positive predictive value	0.500
Negative predictive value	0.796
Positive likelihood radio	1.018
Negative likelihood radio	0.261

### Training and validation of machine learning models

**Figure 9:**
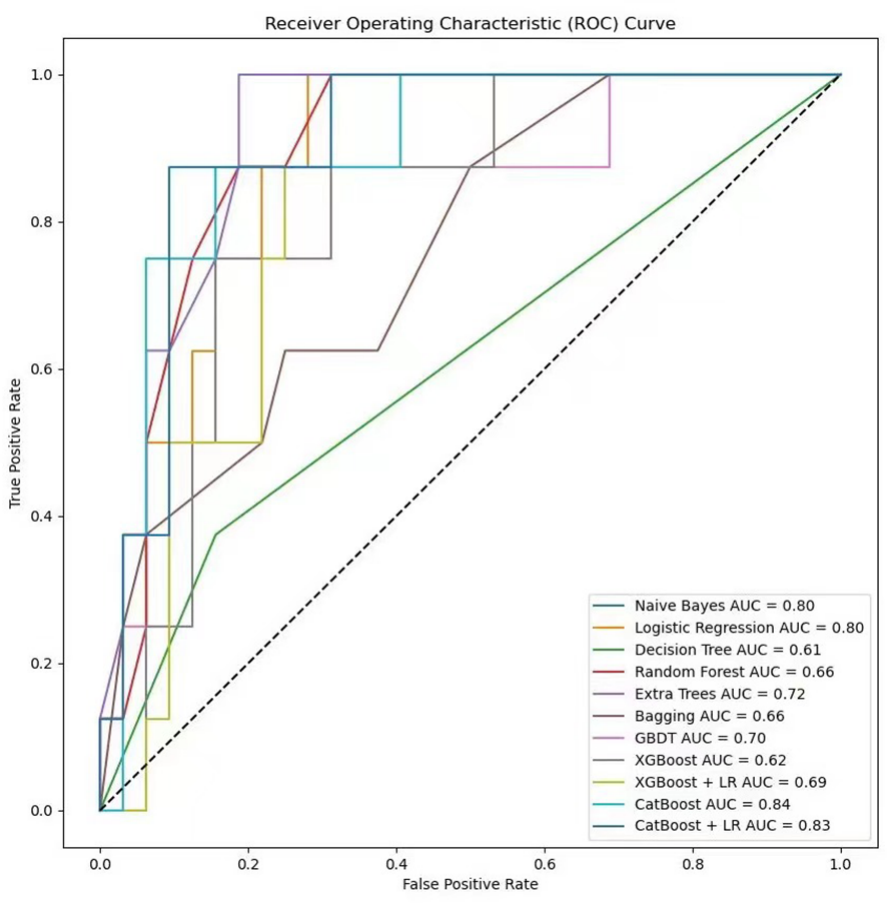
Comparison of ROC curves in machine learning models.

**Figure 10:**
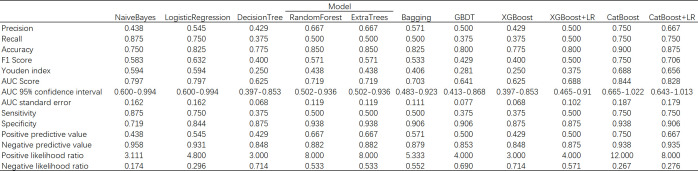
Comparison table of machine learning model performance.

**Figure 11:**
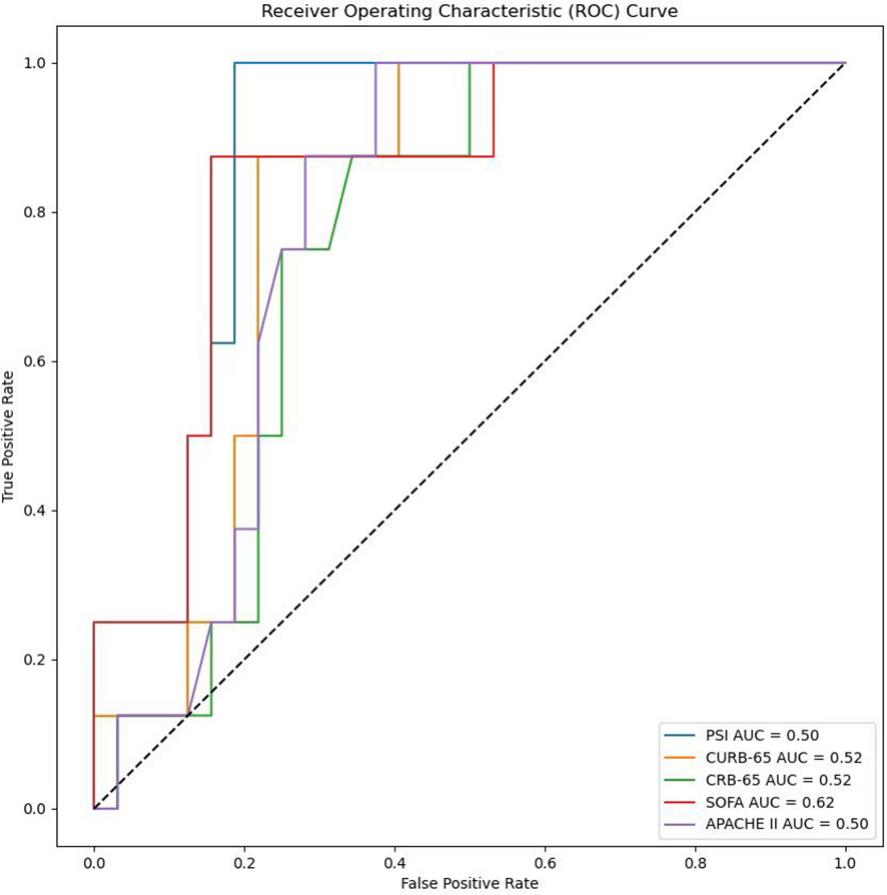
Comparison chart of ROC curves with different clinical scores.

In the process of using machine learning methods to predict the 28-day mortality of SCAP patients, eleven models including Naive Bayes, Logistic Regression, Decision Tree, Random Forest, Extra Trees, Bagging, Gradient Boosting (GBDT), XGBoost (XGB), XGBoost combined with Logistic Regression (XGB+LR), CatBoost, and CatBoost combined with Logistic Regression (CatBoost+LR) were used for prediction. The predictive performance of various models on the test dataset was evaluated using the AUC metric. As shown in **Figure 9**, both Naive Bayes and Logistic Regression with high AUC values of 0.80 indicated good classification performance. In contrast, the AUC value of the Decision Tree model was only 0.61, suggesting its performance was relatively poor. The Random Forest and Bagging models both with the AUC values of 0.66 displayed moderate performance. The Extra Trees model that surpassed some other models with an AUC value of 0.72 demonstrated good performance. The AUC value of GBDT was 0.70, indicating reasonable performance. The AUC value of XGBoost model when used alone was 0.62. Notwithstanding, when combined with LR, its AUC value increased to 0.69. The CatBoost model performed the best among all models presented, with AUC value as high as 0.84. However, when CatBoost was combined with LR, the AUC value slightly decreased to 0.83. Although there was a slight reduction, it still exhibited strong classification ability. **Figure 10** provides a detailed comparison of the performance metrics of each model on the test dataset, including Precision, Recall, Accuracy, F1 score, AUC score, AUC 95% confidence interval, AUC standard error, Youden index, Sensitivity, Specificity, Positive predictive value, Negative predictive value, Positive likelihood ratio and Negative likelihood ratio, totaling 14 evaluation metrics. To further compare the performance of the CatBoost model with the pneumonia score model (PSI, CURB-65, CRB-65, SOFA score,and APACHE II score), the ROC curve was plotted. As shown in **Figure 11**, the AUC value of PSI is 0.50, CURB-65 is 0.52, CRB-65 is 0.52, SOFA is 0.5 and APACHE II is 0.50. These results have indicated that the performance of these traditional pneumonia scoring models is not as effective as the CatBoost model, which shows higher predictive accuracy with AUC value of 0.844.

### Interpretation and evaluation of machine learning models

**Figure 12:**
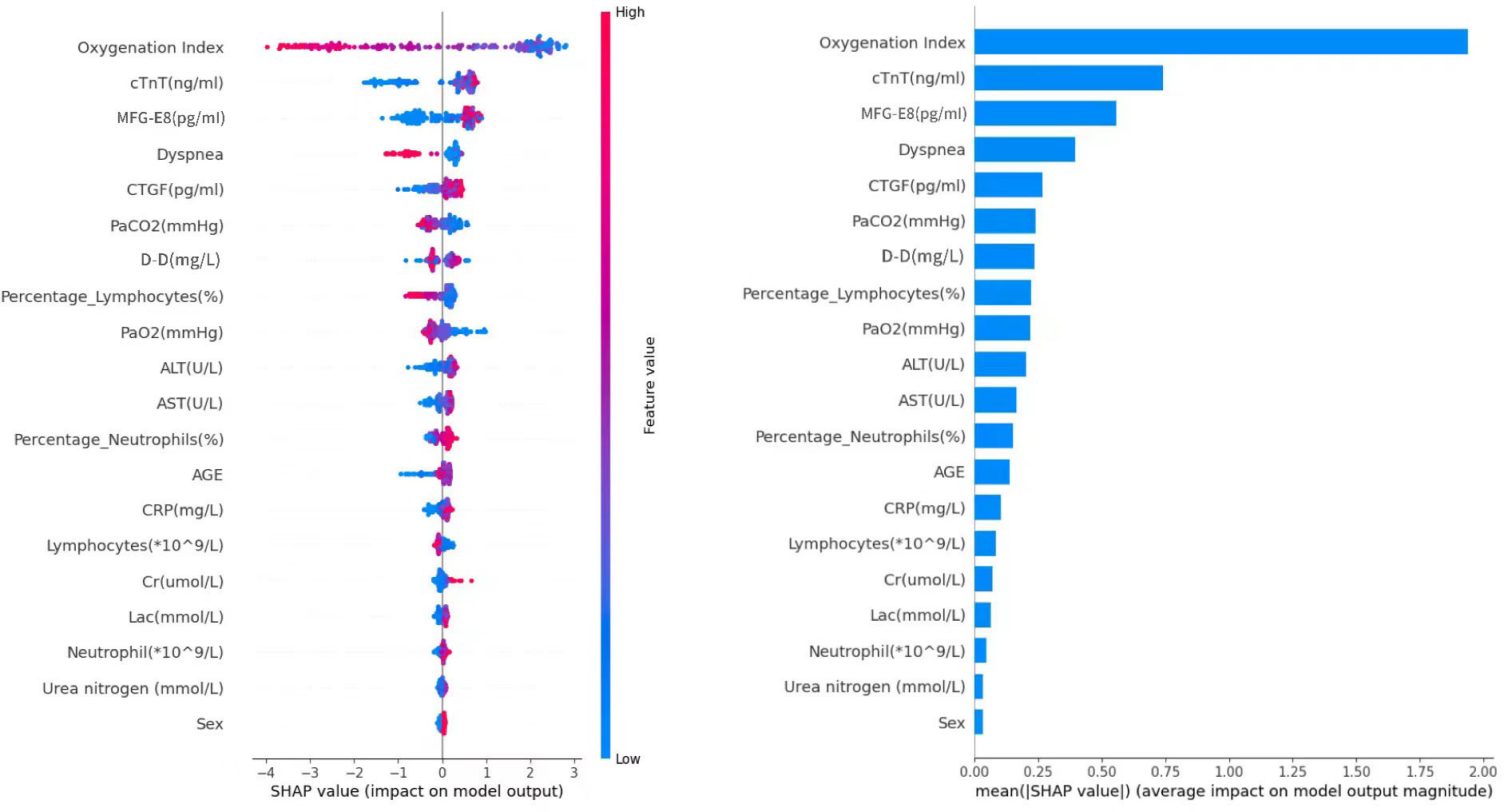
SHAP value analysis of feature importance.

SHAP values (SHapley Additive exPlanations) represent the contribution of specific features to a given model’s prediction. The x-axis in the graph represents the SHAP values, where positive and negative values signify the contribution of increasing or decreasing feature values to the model’s prediction, respectively. Positive values (to the right in the graph) imply that increasing feature values tend to push the prediction towards the positive class, while negative values (to the left in the graph) indicate a tendency towards negative class prediction. Based on the SHAP algorithm, the feature importance ranking explanation of the CatBoost model (**Figure 12**) demonstrates that Oxygenation Index, cTnT, MFG-E8, Dyspnea, CTGF, PaCO2, D-D, Percentage_Lymphocytes, PaO2, and ALT are the most influential features in predicting the results in the CatBoost model.

## Discussion

In this study, the levels of two key biomarkers—CTGF and MFG-E8—in patients with SCAP are significantly elevated compared to healthy controls. Compared to the CAP group, the SCAP group has lower levels of MFG-E8 and higher levels of CTGF. Besides, among SCAP patients, those in the deceased group show elevated levels of CTGF and lowered levels of MFG-E8 compared to the surviving group. These findings have stressed the potential application of these two biomarkers in the diagnosis and prognostic evaluation of SCAP. The results provide clinicians with critical biological information to help identify SCAP patients during diagnosis and offer researchers new directions to explore the specific roles of these biomarkers in the progression of SCAP.

Among SCAP patients, those in the deceased group have elevated levels of CTGF compared to the surviving group, potentially reflecting the role of CTGF in enhancing fibrosis processes and lung injury (24, 48, 49). Research indicates that CTGF is a pro-inflammatory factor regulated negatively by miR-26a-5p, thereby influencing inflammation and tissue repair mechanisms (48). The upregulation of CTGF in pulmonary fibrosis suggests its association with worsening lung conditions. Therefore, CTGF, by directly participating in lung tissue repair and fibrosis, offers more accurate prediction of disease progression compared to other biomarkers by presenting unique advantages and therapeutic potential. MFG-E8, which differs from other biomarkers in predicting SCAP due to its unique role in regulating inflammatory responses and promoting tissue repair (28, 50–52), can reduce neutrophil infiltration and promote the clearance of apoptotic cells, thus preventing excessive inflammatory damage and enhancing lung healing processes. This dual action, particularly in conditions such as ALI and sepsis where MFG-E8 has been shown to reduce inflammation and organ damage, has further highlighted its potential for more targeted and effective treatment strategies in SCAP, even potentially improving patient outcomes (28, 50, 51, 53). These reasons underscore the potential of CTGF and MFG-E8 in predicting mortality in patients with SCAP. Compared to CAP, SCAP patients have higher levels of CTGF. The differences in CTGF and MFG-E8 levels between SCAP and CAP patients reveal the pathophysiology of these two conditions. SCAP patients with elevated levels of CTGF indicate more severe inflammatory responses and active tissue remodeling processes. CTGF, a multifunctional protein involved in tissue repair and fibrosis, suggests that SCAP is associated with intense inflammation, prompting increased CTGF production as the body attempts to repair damaged tissues. Furthermore, the higher CTGF may mean a tendency towards fibrosis, reflecting significant remodeling of lung tissue that could lead to long-term complications. SCAP patients with lower levels of MFG-E8 suggest a failure in the resolution phase of inflammation, which is critical for tissue repair and the clearance of apoptotic cells. This impairment in MFG-E8 levels may lead to prolonged inflammation and subsequent tissue damage, thus contributing to the overall severity of SCAP. Consequently, the inability to effectively resolve inflammation increases the risk of complications, such as ARDS and lung fibrosis, which clearly stress the importance of MFG-E8 in the pathophysiology of SCAP. Overall, the observed variations in CTGF and MFG-E8 levels underscore distinct pathophysiological mechanisms, with elevated CTGF suggesting a robust inflammatory and fibrotic response, while reduced MFG-E8 points to impaired resolution of inflammation, emphasizing the need for targeted interventions to improve patient outcomes.

In this study, a nomogram model and 11 machine learning algorithms were used to predict the overall survival rate of SCAP patients, with 14 scoring methods for evaluation. Besides, the comparative analysis was made to determine the best algorithm against the nomogram model, which was necessary to ensure the selection of the optimal model for the specific management of SCAP patients. Compared to the traditional nomogram model, the CatBoost model in machine learning provided a more personalized and reliable method for predicting the 28-day mortality risk of SCAP patients. Key predictive features identified in the CatBoost model encompassed the Oxygenation Index, indicating respiratory function; cTnT, reflecting cardiac stress; MFG-E8, whose lower levels in deceased patients suggested a protective role against inflammation; dyspnea, correlating with disease severity; CTGF, associated with fibrosis and poor prognosis and PaCO2, indicating respiratory failure. The CatBoost model effectively utilized these features to enhance predictive accuracy by identifying complex patterns in the data, with SHAP analysis revealing how each feature influenced mortality predictions. CTGF and MFG-E8,serving as potential biomarkers for assessing SCAP severity and prognosis, can offer valuable information for risk stratification and clinical decision-making. By integrating them with predictive models aids in identifying high-risk patients, more personalized treatment strategies and improved patient outcomes will be obtained. In short, the identified features are crucial for predicting mortality in SCAP patients, and further research into these biomarkers could deepen our understanding of their roles in respiratory conditions. By virtue of the high complexity and generalization ability of machine learning, these models can handle and analyze large volumes of clinical data to identify the subtle and complex patterns that affect patient prognosis. They can not only make predictions based on a wide range of variables but also continuously optimize prediction accuracy as new data is inputted, thereby providing clinicians with powerful decision-support tools to optimize patient management and improve the timeliness and specificity of clinical interventions.

On the other hand, while machine learning models have unique advantages in handling big data and recognizing complex patterns, they usually require specialized knowledge to build and interpret and their black-box nature can make the decision-making process difficult for non-experts to understand. Additionally, implementing and running machine learning models typically relies on computer or cloud infrastructure, which may not be practical in resource-limited settings or situations requiring rapid decision-making.

Overall, the study underscores the importance of CTGF and MFG-E8 as potential biomarkers for predicting outcomes in SCAP patients, impacting personalized treatment strategies in several ways. Elevated CTGF and reduced MFG-E8 levels can facilitate early diagnosis and risk stratification, allowing clinicians to provide aggressive treatment for high-risk individuals and effectively allocate resources. Additionally, targeting CTGF through anti-fibrotic therapies may mitigate lung damage, while administering recombinant MFG-E8 could enhance tissue repair and immune regulation. The deployment of machine learning models, particularly CatBoost, can refine patient outcome predictions and adapt treatment plans in real-time based on evolving clinical data. To translate these findings into clinical practice, future steps include conducting multicenter validation studies, developing standardized assays for biomarker measurement, designing clinical trials for biomarker-driven therapies, integrating machine learning into electronic health records for real-time decision support, and working towards regulatory approval of CTGF and MFG-E8 in clinical guidelines. By addressing these areas, the study’s findings can significantly enhance personalized treatment approaches for SCAP patients and facilitate their implementation in clinical settings.

However, the limitations of this study should be noted. Firstly, the sample size is limited. All subjects are from a single center, which may affect the generalizability of the results. Therefore, the future research should involve broader and more diverse samples from multiple centers to strengthen the validation of the current findings. Secondly, although elevated levels of MFG-E8 and CTGF have been observed in SCAP patients, the specific mechanisms behind these elevations are not yet clear, which require further laboratory studies to explore. Thirdly, this study has only measured serum levels of MFG-E8 and CTGF, but without considering the distribution of these biomarkers in local tissues. Future plans should include measuring MFG-E8 and CTGF levels in bronchoalveolar lavage fluid to gain a more comprehensive understanding. Fourthly, since it’s an epidemiological study, the causality has not been established. Further *in vivo* and *in vitro* experiments are needed to verify these results. Additionally, this study did not consider the temporal changes in CTGF and MFG-E8 levels and their relationship with SCAP progression. But time-series data analyses may provide more information about the roles of these biomarkers in the disease process. Despite the impressive performance of the CatBoost model in predicting 28-day mortality in SCAP patients in this study, it is essential to recognize that the model’s high predictive ability may be limited to specific patient populations and clinical practice environments. It means the model’s predictive accuracy and applicability may vary in different regions or healthcare systems, as there are differences in medical resources, treatment methods, patient demographics and disease prevalence trends across regions. Therefore, the extensive validation of the CatBoost model’s effectiveness is not only a crucial step in improving the model’s predictive performance, but also a vital aspect of advancing personalized medicine and precision healthcare.

## Data Availability

The original contributions presented in the study are included in the article/supplementary material. Further inquiries can be directed to the corresponding author.
